# VOCs-Mediated Location of Olive Fly Larvae by the Braconid Parasitoid* Psyttalia concolor*: A Multivariate Comparison among VOC Bouquets from Three Olive Cultivars

**DOI:** 10.1155/2016/7827615

**Published:** 2016-02-18

**Authors:** Giulia Giunti, Giovanni Benelli, Giuseppe Conte, Marcello Mele, Giovanni Caruso, Riccardo Gucci, Guido Flamini, Angelo Canale

**Affiliations:** ^1^Department of Agriculture, Food and Environment, University of Pisa, Via del Borghetto 80, 56124 Pisa, Italy; ^2^Interdepartmental Research Center Nutrafood “Nutraceuticals and Food for Health”, University of Pisa, Pisa, Italy; ^3^Department of Pharmacy, University of Pisa, Via Bonanno 6, 56126 Pisa, Italy

## Abstract

Herbivorous activity induces plant indirect defenses, as the emission of herbivorous-induced plant volatiles (HIPVs), which could be used by parasitoids for host location.* Psyttalia concolor* is a larval pupal endoparasitoid, attacking a number of tephritid flies including* B. oleae. *In this research, we investigated the olfactory cues routing host location behavior of* P. concolor *towards* B. oleae* larvae infesting three different olive cultivars. VOCs from infested and healthy fruits were identified using GC-MS analyses. In two-choice behavioral assays,* P. concolor* females preferred infested olive cues, which also evoked ovipositional probing by female wasps. GC-MS analysis showed qualitative and quantitative differences among volatiles emitted by infested and healthy olives. Volatile emissions were peculiar for each cultivar analyzed. Two putative HIPVs were detected in infested fruits, regardless of the cultivar, the monoterpene (*E*)-*β*-ocimene, and the sesquiterpene (*E-E*)-*α*-farnesene. Our study adds basic knowledge to the behavioral ecology of* P. concolor*. From an applied point of view, the field application of the above-mentioned VOCs may help to enhance effectiveness of biological control programs and parasitoid mass-rearing techniques.

## 1. Introduction

The olive tree (*Olea europea*) is an economically important crop in the Mediterranean basin, holding about 98% of world's olive groves [1]. In the last decades, olive crop was also widespread in novel regions, such as China, Brazil, and South Africa, increasing olive production up to 20.4 million tons in 2013, one of the highest production levels ever recorded. On the other hand, the olive crop spread has determined diffusion of the most devastating insect pest of olives, the olive fruit fly,* Bactrocera oleae* (Rossi) (Diptera: Tephritidae). Its diffusion occurred in the Mediterranean regions for over 2000 years, and, more recently, in California olive grows [2].* B. oleae*, is a monophagous pest, feeding exclusively on* Olea* species. Olive fruit fly females lay an egg under the fruit surface; thus the larvae develop inside olive fruits until they open an exit hole before pupate. On table olive groves the oviposition puncture leads to a serious reduction of crop value, while exit holes and pulp degradation can determine a quality and quantity loss of olive oil production.* B. oleae* infestation can reduce oil yield [3, 4], alter several quality parameters (e.g., acidity, peroxide value, UV absorbance) [1, 5–7], and even negatively impact chemical composition, which determine oil taste and flavor [1, 6–10]. Volatiles profiles are known to be influenced by abiotic factors [11], but also* B. oleae* infestation could induce critical changes of volatile emissions [12].

Herbivorous feeding activity is known to induce a variety of biochemical changes in plants. It is well known that plants respond to herbivores' presence activating their defense system [13], but they can also trigger indirect defenses, as the emission of herbivorous-induced plant volatiles (HIPVs, hereafter) [14, 15]. The role of kairomones on parasitoid host location has been widely investigated [16–18] and it has been demonstrated that many plants rely on volatile signals induced by phytophagous feeding to attract their natural enemies [14, 19, 20]. Moreover, despite the evidence about the influence of* B. oleae* infestation on the quality and the quantity of volatile compounds emitted in olive oils [7, 8], no information is available to assess the presence of HIPVs produced by infested olive fruits. However, differential emissions have been already proved for Tephritidae-infested and healthy fruits [21–24], highlighting the production of several HIPVs able to evoke electrophysiological and behavioral responses in parasitoid wasps [23, 24].

*Psyttalia concolor* (Szépligeti) (Hymenoptera: Braconidae) is a koinobiont larval pupal endoparasitoid, able to parasitize at least fourteen tephritids on different wild and/or cultivated plants, including* B. oleae* and* Ceratitis capitata* (Wiedemann), the Mediterranean fruit fly [25].* P. concolor* females rely on a number of stimuli to successfully locate their host. Indeed, female wasps are able to distinguish between infested and healthy fruit, preferring the first one, even if just olfactory cues are provided [23]. In addition, it was demonstrated that apple and peach fruits infested by* C. capitata* larvae emitted peculiar volatiles, recognized by* P. concolor* wasps and able to attract selectively mated females [23]. HIPVs from apple and peach fruits are also able to attract and prolong the time spent performing searching behavior in* P. concolor* virgin males, probably raising their chances to locate receptive females nearby host microhabitat [26]. Furthermore, even synthetic blends reproducing infested peaches or apples were found able to be attractive for* P. concolor* mated females and virgin males [23, 26].

In this research, we investigated the importance of olfactory cues used by* P. concolor* females to locate their host microhabitat. We hypothesize that the HIPVs from* B. oleae*-infested olive fruits may play a pivotal role in affecting* P. concolor* host location, as described for the same parasitoid on a different tephritid host [23]. Olive fruits from three different cultivars were tested to determine parasitoid attractiveness and volatile organic compounds (VOCs) emissions: cv. Frantoio and cv. Leccino (traditionally cultivated in Italy) and cv. Arbequina (typical of Spanish olive groves). Firstly we evaluated females' preferences among healthy and infested fruits in two-choice bioassay, providing both visual and olfactory cues or olfactory stimuli alone, in order to evaluate the magnitude of volatiles attractiveness. Subsequently, volatiles emitted by healthy and infested olive fruits were SPME-sampled and analyzed by gas chromatography-mass spectrometry (GC-MS) to estimate differentially emissions attributable to herbivores' activity and to indicate possible HIPVs.

## 2. Material and Methods

### 2.1. Parasitoid Rearing

*P. concolor *wasps were reared as described by Canale and Benelli [25]. Insects were maintained in Pisa Laboratory under controlled conditions (22°C ± 1, 50–60% relative humidity and natural photoperiod) during their entire life. Adult parasitoids were allowed to emerge in transparent Plexiglas tubes (diameter 40 cm, length 50 cm) into which 1500 adults were introduced (male : female sex ratio 0.3–0.5). To obtain pupae, from which the adult emerged, a nylon mesh bag containing around 700 third instar* C. capitata* larvae was posed into a cage and exposed to* P. concolor* wasps for 20 minutes. Parasitized pupae were placed into smaller Plexiglas cages (diameter 20 cm, height 30 cm) and there* P. concolor* adults were allowed to emerge at a density of 50 specimens per cage (males : females sex ratio 0.3). Insects were stored at laboratory conditions [22 ± 1°C, 50 ± 5% relative humidity and 16 : 8 (L : D) photoperiod] for 7 days after the parasitoids' emergence to allow mating before testing. Adult insects were fed on a semisolid diet (honey mixed with pollen) and with water ad libitum.

### 2.2. Plant Material

Olive fruits from three different cultivars (Frantoio, Leccino, and Arbequina) were used for behavioral assay and GC-MS analysis. Olives were collected on September 15, 2014, in Tuscan olive groves [Frantoio and Leccino from Torrita di Siena (43°15′49.86′′N, 11°78′96.58′′E) and Arbequina from Rapolano Terme (43°27′70.97′′N, 11°60′70.98′′E)] from 5–3-year olive trees. Healthy or infested olives from each cultivar were collected manually, stored into glass jars (diameter 10 cm, length 20 cm) and transferred to laboratory conditions within 3 hours. The fruits were firstly divided according to the maturation index (MI), whereby the skin and flesh colors were scored to a 0 to 7 scale [27], and olives with MI from 2 to 7 were discharged. Among infested olives, we selected the ones attacked second or early third instar larvae, with no exit holes on the olive surface. Healthy fruits were selected avoiding crushed and naturally damaged ones.

Before being tested fruits were stored at laboratory condition for 2–5 days. All olives used for both behavioral and GC-MS tests were subsequently dissected to check the presence or the absence of* B. oleae* larvae inside the fruits.

### 2.3. Effect of Olfactory Cues from Infested Olives on Parasitoid Attractiveness

Bioassays were conducted using the still air arena described by Benelli et al. [28]. A Plexiglas unit (150 × 150 × 30 mm) was covered on the top with a removable glass panel to create the arena. The unit presents a circular chamber (diameter 40 mm) in the center to release the specimen and two other identical chambers connected with linear paths (length 20 mm; width 10 mm) where the stimuli were allocated.

To assess if infested olives are attractive for* P. concolor*, in a first experiment, mated females were allowed to choose among three healthy or infested olive fruits of each cultivar. In addition, to investigate the role of olfactory stimuli in leading parasitoid host location, a second experiment was designed. As in the first experiment three infested or healthy fruits were placed into the test chambers, but a piece of filter paper was posed ahead of the fruits to avoid visual contact with the parasitoid female released in the central chamber.

A replicate starts when a wasp was gently transferred to the released chamber and observed for 8 min. A wasp was considered to have to choose a cue when it remains in the same chamber for at least 20 s actively searching for a host and the replicate was considered complete when the wasp left the chamber. Wasps that show no choice after 7 min were not considered. With each new wasp, the arena was rotated 90° and the relative position of cues was randomized. After each assay the arena was cleaned washing firstly with warm water, then rinsed in a water bath with mild soap, subsequently washed with hot water, and eventually cleaned with distilled water [29]. 30 mated females were tested for each treatment. For each bioassay the (i) latency time (time elapsing from the start of the replicate and the effective choice), (ii) female's first choice, (iii) time spent on the chosen chamber, (iv) number of antennal drumming series (performed in close proximity of the stimulus), and (v) number of oviposition attempt (performed on the fruits or on the filter paper surface) were recorded.

For each choice-test, a likelihood chi-square test with Yates correction (with *α* = 0.05) was used to compare the proportion of parasitoids choosing a given cue [30]. The other measured variables were analyzed in JMP 7® by using a general linear model with one fixed factor (i.e., the treatment).

### 2.4. Effect of B. oleae Infestation on VOCs Production

Supelco (Bellefonte, PA, USA) SPME devices coated with polydimethylsiloxane (PDMS, 100 *μ*m) were used to sample the headspace of three olive fruits (healthy or infested by* B. oleae*) inserted into a 30 mL glass vial and allowed to equilibrate for 30 min. SPME sampling was performed using the same new fibre, preconditioned according to the manufacturer instructions, for all the analyses. Sampling was accomplished in an air-conditioned room (25 ± 1°C) to guarantee a stable temperature. After the equilibration time, the fibre was exposed to the headspace for 30 min. Once sampling was finished, the fibre was withdrawn into the needle and transferred to the injection port of the GC-MS system. All the SPME sampling and desorption conditions were identical for all the samples. Furthermore, blanks were performed before each first SPME extraction and randomly repeated during each series. For each cultivar, three replicates (either containing three olives) for both infested and healthy fruits were provided. Quantitative comparisons of relative peaks areas were performed between the same chemicals in the different samples.

Gas chromatography/electron impact mass spectroscopy (GC-EIMS) analyses were performed with a Varian CP-3800 gas chromatograph equipped with a DB-5 capillary column (30 m × 0.25 mm; coating thickness = 0.25 *μ*m) and a Varian Saturn 2000 ion trap mass detector (emission current: 10 microamps; count threshold: 1 count; multiplier offset: 0 volts; scan time: 1.00 second; prescan ionization time: 100 microseconds; scan mass range: 20–300 *m*/*z*; ionization mode: EI). The following analytical conditions were used: injector and transfer line temperature at 250 and 240°C, respectively; oven temperature programmed from 60 to 240°C at 3°C min^−1^; carrier gas, helium, at 1 mL min^−1^; splitless injection. Identification of the constituents was based on comparison of the retention times (RT) with those of pure compounds, comparing their linear retention indices (LRI) relative to the series of* n*-hydrocarbons and on computer matching against commercial (NIST 98 and ADAMS) and homemade library mass spectra built from pure substances and components of known oils and MS literature data [31–35].

For each compound and chemical class, the area integration report was transformed into log values, before statistical analysis. The normal distribution of data was checked using Shapiro-Wilk test. To evaluate differences in volatile emissions between infested and healthy fruits of the three cultivars, the variance was analyzed with JMP 7 by using a general linear model with one fixed factor (i.e., fruit health status). In addition a general linear model with two factors, health status and cultivar, was performed: *y*_*j*_ = *μ* + *I*_*j*_ + *C*_*j*_ + (*H*_*j*_ × *C*_*j*_) + *e*_*j*_, in which *y*_*j*_ is the observation, *μ* the overall mean, *I*_*j*_ the fruit infestation status (*j* = 1-2), *C*_*j*_ the cultivar (*j* = 1–3), *I*_*j*_ × *C*_*j*_ the interaction infestation status × cultivar, and *e*_*j*_ the residual error.

Principal Component Analysis (PCA) was achieved on normalized values of each VOC to derive different variables (principal components) that summarize the original data. PCA analysis was performed using JMP software. PCA calculated linear combination of the original data extracting eigenvalues and eigenvectors of a correlation matrix of volatiles' areas and highlighted principal components, the orthogonal and linear combination of the original variables. Two-dimensional score plots were created to determine if volatiles from different olive fruit cultivar or with different infestation degree could be clustered into classes. Then, a Multifactorial Analysis (MFA) was performed to assess common factors explaining volatiles' variability using a maximum likelihood estimation procedure and a VARIMAX orthogonal rotation technique by JMP. Scores of common factors were calculated as described by Macciotta et al. [36]. Furthermore, factors scores were analyzed using a general linear model with infestation status and cultivars as fixed factors, to enlighten the relationship between a common factor and the various treatments. Discriminant analysis, also performed using JMP software, used different volatiles, which can be highly correlated to a given fixed variable (i.e., infestation status), as a set of independent variables. A step-wise method was used to select a set of independent variables with *R* ^2^ > 0.1. The ratio (Wilks's lambda) between the generalized within-category dispersion and the total dispersion was considered [37].

## 3. Results

### 3.1. Effect of Olfactory Cues from Infested Olives on Parasitoid Attractiveness

**Figure 1:**
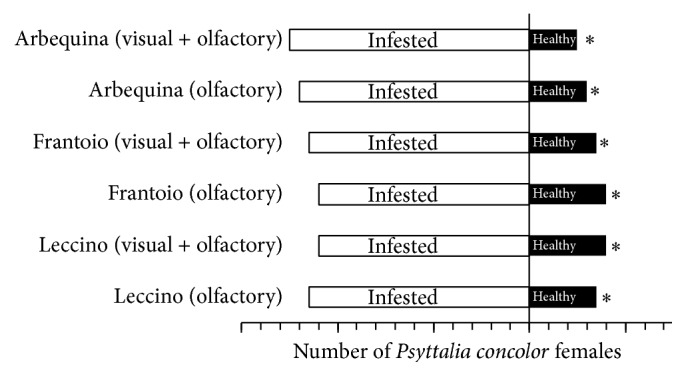
Attractiveness of* Bactrocera oleae*-infested fruits towards* Psyttalia concolor* mated females: effect of visual and olfactory. Two-choice bioassays were conducted in a still air arena with olive fruits, infested or not by olive fruit fly larvae, providing visual and olfactory stimuli associated or only olfactory cues. Thirty wasps were tested in each bioassay. For each test, asterisks indicate significant differences in the number of wasps choosing different cue (*χ* ^2^ test with Yates correction, *P* < 0.05).

*P. concolor* mated females showed significant preferences for infested fruits over healthy ones when both visual and olfactory cues were provided (Arbequina: *χ* ^2^ = 10.8333, df = 1, *P* = 0.0010; Frantoio: *χ* ^2^ = 8.5667, df = 1, *P* = 0.0034; Leccino: *χ* ^2^ = 6.5667, df = 1, *P* = 0.0104) (Figure 1). No significant differences were recorded for latency times and times spent on the chosen chamber (Table 1), while wasps which had preferred infested fruits of Frantoio and Leccino varieties performed a greater number of drumming series on infested fruits (Table 2). No oviposition attempts were noted when females visited healthy olives, conversely to oviposition and probing behaviors recorded in infested fruits of all varieties.

Infested olives were positively located and chosen even in absence of visual stimuli, when fruits were hidden by filter paper. Indeed,* P. concolor* females preferred to prospect chambers containing infested fruits over healthy ones (Arbequina: *χ* ^2^ = 13.3667, df = 1, *P* = 0.0003; Frantoio: *χ* ^2^ = 6.5667, df = 1, *P* = 0.0104; Leccino: *χ* ^2^ = 8.5667, df = 1, *P* = 0.0034) (Figure 1). No significant differences for latency times were found, but* P. concolor* females spent longer times in the chamber with Arbequina infested fruits than with healthy Arbequina olives (Table 1). Indeed when Arbequina or Leccino infested fruit odor was preferred by tested wasps, females accomplished a higher number of drumming series (Table 2). No oviposition attempts were recorded for wasps choosing healthy fruit chamber, while, interestingly, some wasps were noted to perform probing behavior in the filter paper or in the glass walls of the chamber containing infested olives.

### 3.2. Effect of B. oleae Infestation on VOCs Production of Olive Fruits from Different Cultivar

GC-MS analysis identified over 100 different volatile compounds. Differential emissions attributable to herbivore activity were found for all cultivars. In detail, we found 6 compounds significantly increased in infested olives and 6 volatiles were exclusively produced by infested fruits of Arbequina variety (Supplementary Table S1; see Supplementary Material available online at http://dx.doi.org/10.1155/2016/7827615). In Frantoio, 3 compounds were exclusive and 3 increased and one decreased in infested olives (Supplementary Table S2), while in Leccino we found 4 compounds increasing and 4 exclusively present in infested fruits (Supplementary Table S3). In detail, the three cultivars present 2 common VOCs prevalently produced by infested olive fruits: (*E*)-*β*-ocimene and (*E-E*)-*α*-farnesene. Among chemical classes, monoterpenes hydrocarbons increased in all cultivars (Arbequina: *F* = 8.0698, df = 1, *P* = 0.0468; Frantoio: *F* = 35.4752, df = 1, *P* = 0.0040; Leccino *F* = 14.3467, df = 1, *P* = 0.0193). Indeed, Arbequina infested fruit showed different emissions of ketones (*F* = 10387.18, df = 1, *P* < 0.0001) and sesquiterpenes hydrocarbons (*F* = 24.1958, df = 1, *P* = 0.0079), while Frantoio increased monoterpenes oxygenated (*F* = 17.5960, df = 1, *P* = 0.0138) and aromatic hydrocarbons (*F* = 50.5679, df = 1, *P* = 0.0021). From two factors general linear model, 36 compounds, and 6 chemical classes were noted to be significant for at least one factor (Supplementary Table S4).

**Figure 2:**
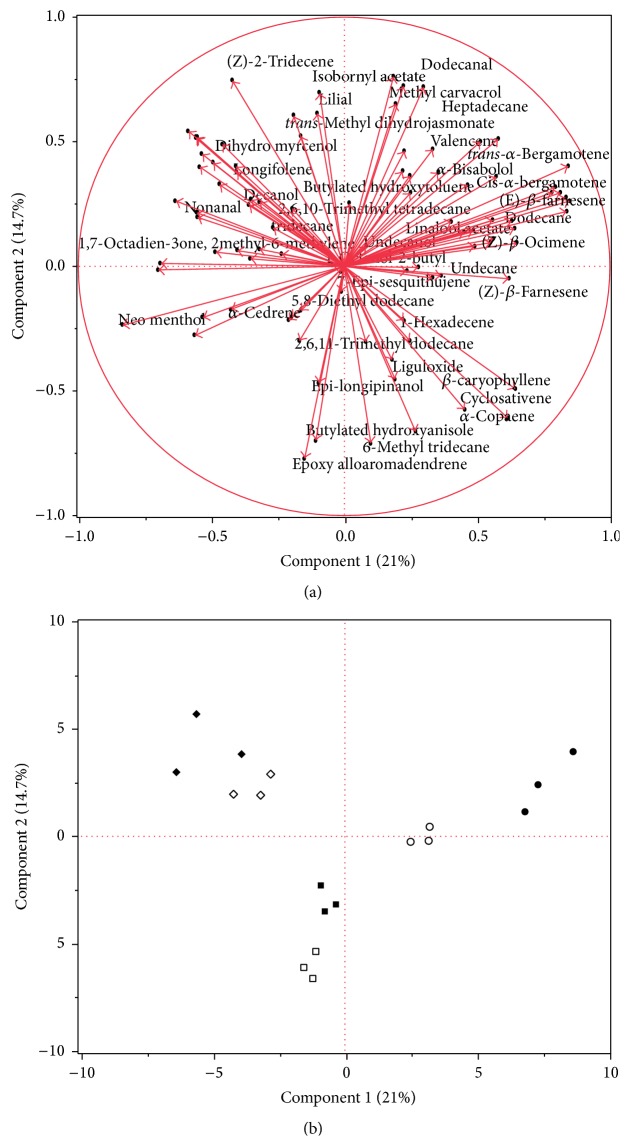
Principal Component Analysis (PCA) of volatile profiles from infested and healthy fruits of three different olive cultivars. (a) PCA loading plot, showing volatile correlations with the first and second principal component; (b) PCA score plot, highlighting cluster of volatiles attributable to cultivar or infestation status. ● Arbequina infested fruits; ○ Arbequina healthy fruits; ■ Frantoio infested fruits; □ Frantoio healthy fruits; ◆ Leccino infested fruits; ◊ Leccino healthy fruits.

**Figure 3:**
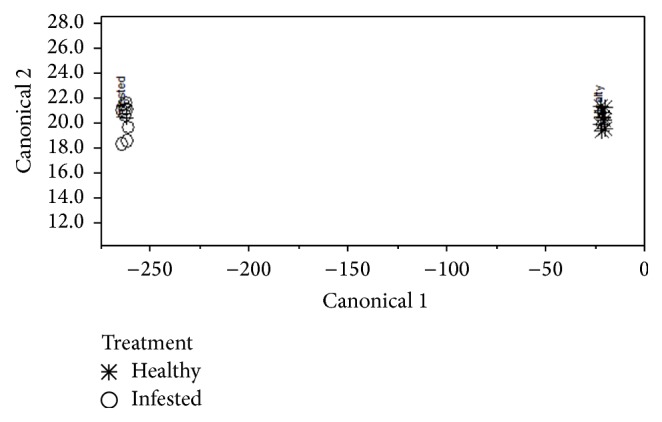
Canonical plot from discriminant analysis showing compounds highly correlated with Canonical 1 variable representing the infestation status in olive cultivars.

Furthermore, PCA followed by discriminant analysis allowed a more precise partition of cultivar and infestation effects on volatile emission from fruits. The Kaiser coefficient was around 1.00 since no correlations existed between the majorities of the compounds. Six principal components were analyzed, explaining 66.730% of variation (Table 3).  Figure 2 shows PCA results and two-dimensional score plots were created to highlight different clusters relative to different olive fruit cultivar and different infestation status (Figure 3). Eigenvectors of single VOCs are provided in Supplementary Table S5 and rotated factor patterns in Supplementary Table S6. The rotated factors with an eigenvector of at least ±0.5 were marked in bold and considered for the following analysis. A two-way general linear model was provided to understand which sources of variation had a significant effect on the six analyzed factors, as reported by Supplementary Table S7. On this basis, we labeled the six factors as: Factor 1 “Infestation,” Factor 2 “cv. Frantoio,” Factor 3 “Italian Varieties,” Factor 4 “Infestation cv. Leccino,” Factor 5 “cv. Arbequina,” and Factor 6 “cv. Leccino.” Discriminant analysis was provided for one source of the variations (i.e., infestation status). Wilks' Lambda test showed a *P* value < 0.0001 and no misclassified variables were recorded. Step-wise method emphasized 11 variables highly correlated to infestation status (Table 4). Two VOCs (6-methyl-3-methylene-5-hepten-2-one and 2,6,11-trimethyldodecane) resulted positively correlated with Canonical 1, representing compounds typically associated with healthy olives, while the other 9 compounds were expression of infested status (Figure 3).

## 4. Discussion

Olfactory stimuli from host-infested fruits are known to be essential during host location behavior for many braconids, including species attacking larval stages [24, 38–43]. For* P. concolor*, the presence of chemical compounds was demonstrated produced by* C. capitata*-infested apples and peaches able to attract both mated females and virgin males [23, 26]. The evidence that olfactory cues from infested fruits evoke behavioral responses from mated* P. concolor* females and of the presence of compounds that were produced exclusively or in higher amount by infested olives supports our hypothesis that VOCs could act as short-range attractant, playing a key role during host-seeking also in this tritrophic system.* P. concolor* were attracted preferentially by infested olives, both when visual stimuli were provided or not, suggesting that the presence of feeding larvae inside the fruit is crucial for host location. Indeed, oviposition behavior was performed from females just when they chose infested fruit stimuli. Interestingly ovipositor probing responses were performed also by* P. concolor* females which did not come directly in contact with olive fruits, but only sensing infested olive odors. This behavior, already described for* P. concolor* females attracted by some synthetic HIPVs [23], is uncommon among parasitic wasps, since usually they need an integration of visual and olfactory stimuli to perform a complete host location sequence [21]. Moreover,* P. concolor* females showed probing behaviors on the chamber glass surface in presence of volatile emitted by all the three cultivars, inducing also longer active searching activities, with particular reference to antennal drumming.

To determine whatever change in volatile emissions could explain parasitoid behavior,* B. oleae*-infested and healthy olive fruits were analyzed. Among over 100 VOCs identified by SPME and GC-MS techniques, only two volatiles were found to increase in infested olives in all the three cultivars, a monoterpene, (*E*)-*β*-ocimene, and a sesquiterpene, (*E-E*)-*α*-farnesene, which are already known as constituent of the odors of olive oils and processed table olives [11, 44–46]. However, since (*E*)-*β*-ocimene is attractive to several braconid species, with special reference to* Aphidius* species [47–49] and (*E-E*)-*α*-farnesene which has been demonstrated to attract* Opius dissitus* Muesebeck wasps [50], these two compounds can be considered as putative kairomones for* P. concolor*. Although we observed mainly quantitative changes in volatile emissions among infested and healthy olives, which is not uncommon even in similar tritrophic systems [23, 51, 52], indeed, several plants react to herbivore damages by producing blends of metabolites with changes in number or in their proportions [21, 24].

Moreover, PCA analysis has highlighted that VOCs emissions are peculiar for each cultivar and chemicals, which were differentially emitted after herbivore infestation, changed depending on the olive varieties. Indeed, after multifactorial analysis, we could describe the variability due to baseline healthy cultivar emissions using three different factors (Factor 2, Factor 5, and Factor 6), explaining each one VOC emission of a specific cultivar. In addition, Italian cultivars (cv. Frantoio and cv. Leccino) showed common volatiles explained by Factor 3, which were never produced by the Spanish one (cv. Arbequina). On the other hand, infestation status could be explained for all the three cultivars by a common factor (Factor 1), but we identified also some exclusive compounds which were emitted only by Leccino olives under* B. oleae* infestation (Factor 4). Moreover, Arbequina variety showed to emit differentially the larger number of VOCs. Beside (*E*)-*β*-ocimene and (*E-E*)-*α*-farnesene, Arbequina infested olives increased the emission of other 4 compounds (methyl carvacrol,* n-*tridecane,* trans*-*α*-bergamotene, and* cis*-*β*-farnesene) and produced specifically 6 compounds [(Z)-*β*-ocimene, 2-methyl-6-methylene-1,7-octadien-3-one, cyclosativene, 1-undecanol,* cis*-*α*-bergamotene, and 3,5-di-tert-butylpyrocatechol]. Most of them are known to be common floral compounds, but interestingly some of them are recognized pheromones for several hymenoptera species [53–55], while (Z)-*β*-ocimene is known to be an attractant for the braconid* Diachasmimorpha longicaudata* (Ashmead) [24]. When attacked by* B. oleae*, Leccino cultivar similar to Arbequina increased the production of the floral compound 2-methyl-6-methylene-1,7-octadien-3-one. In addition, Leccino infested olives increased the emission of limonene and exclusively produced 4 monoterpenes (isocineole, *γ* terpinene, dihydromyrcenol, and terpinolene), the majority of which are HIPVs produced by mango fruits, positively tested by Carrasco et al. [24] on* D. longicaudata *wasps. Frantoio cultivar seems to be the less odorant varieties, since it produced only 5 compounds when herbivory attack succeeds. As described for Arbequina, Frantoio infested olives emitted more (*E*)-*β*-ocimene and methyl carvacrol, but they specifically generated [besides (*E-E*)-*α*-farnesene] dihydrocitronellol and heptylcyclohexane, which to the best of our knowledge were never investigated for their attractiveness toward insects. Interestingly, infested Frantoio olives showed to decrease the production of 2,6,11-trimethyldodecane, a peculiar VOC never identified on olives or olive oils. Conversely, the majority of the other identified VOCs are common volatiles emitted by olive oils [11, 44, 56–59], processed table olives [45, 46], leaves [44, 60], and olive fruits, regardless of their infestation status [61].

Hence, the cultivar seems to be the higher source of variation for VOC emissions in olive trees. For this reason, we cannot exclude that other VOCs produced specifically by one cultivar or increased in not all varieties may act as attractant toward* P. concolor* wasps. Indeed, it was demonstrated that also healthy fruits can produce volatiles attractive for parasitic wasps [24], and generalist parasitoid, as* P. concolor*, could be able to perceive cues from non-infested plant to locate host microhabitat. Thus, short-range volatiles produced by the plants or as excretion of the feeding larvae and/or vibrational stimuli from the hosts could be useful to the successful localization [62].

Overall, since HIPVs are known to act as kairomones for several parasitic wasps [24, 63, 64], further researches are needed to assess the activity of the highlighted compounds on parasitoid behavior. Indeed, knowledge about tritrophic system communications has potential implications also on biological control programs [65]. Synthetic kairomones have been already tested in field conditions for parasitoid attraction [64–66], but beside field applications, HIPVs may also be employed to enhance mass-rearing techniques.

## 5. Conclusions

Our results support the hypothesis that chemical cues produced by olive fruits under* B. oleae* attack route the host location behavior of* P. concolor* females, acting as short-range kairomones. Olfactory cues seem to have a key role in host-seeking behavior for two olive cultivars traditionally cultivated in Italy (cv. Frantoio and cv. Leccino) and one Spanish variety (cv. Arbequina). Indeed, behavioral assays have shown that* P. concolor* mated females can perceive the presence of host larvae inside a fruit when visual and olfactory stimuli were provided, but also when visual perception was forbidden. In addition, females choosing fruits infested by* B. oleae* performed longer searching activities, with particular references to antennal drumming series completed on the fruit or in close proximity, and olfactory cues from infested olives evoked ovipositor probing behavior even in absence of direct contact between the parasitoid and the fruits. SPME and GC-MS analysis have also supported the presence of volatiles attributable to herbivore activity which can be indicated as putative HIPVs. In detail, we found 12 volatiles increasing or exclusively emitted by infested Arbequina olives, 5 in Frantoio, and 8 in Leccino ones. Interestingly, the three cultivars showed 2 common VOCs produced as response of* B. oleae* infestation: (*E*)-*β*-ocimene and (*E-E*)-*α*-farnesene. Moreover, PCA and MFA highlighted that the cultivar is a higher source of variation for VOC emissions in olive trees.

Since HIPVs are recognized as kairomones of a number of parasitic wasps [23, 24, 63, 64], further studies are necessary to ensure the behavioral activity of these investigated volatiles toward* P. concolor* parasitoids. Synthetic kairomones may be useful to improve biological control programs, but even techniques for parasitoid mass-rearing. Moreover, even if the efficacy of synthetic kairomonal molecules has been already proved in field conditions [54–66], the role of HIPVs on the foraging behavior of beneficial arthropods in agroecosystems needs to be investigated deeper to enable their safe commercial applications [19].

## Supplementary Material

Tables S1, S2, and S3 present the volatiles emitted by healthy and olive fruit fly-infested fruits from cultivar Arbequina, Frantoio and Leccino, respectively. Table S4 showed the General Linear Model results. (A) Classes of volatiles emitted by healthy and infested olive fruits. (B) Effect test results for the two main variables (Cultivar and Infestation Status) and their interaction (Cultivar∗ Infestation Status).Table S5 provides the Eigenvectors of each volatile compounds emitted by olive fruits for six selected Principal Components. Table S6 highlighted the correlations between volatiles from olive fruits and factors identified after Multi-Factorial Analysis (MFA). Table S7 focuses on the effect tests of two way General Linear Models of Factors obtained after Multi-Factorial analysis (MFA) respect to the main variable (Cultivar and Infestation Status) and their interaction (Cultivar∗ Infestation Status).

## Figures and Tables

**Table 1:** Choice time spent by *Psyttalia concolor* females during searching behavior on healthy and *Bactrocera oleae*-infested olives in two-choice bioassay in still air arena.

Cultivar	Treatment	Infested olives		Healthy olives		*F*	*P* value
		Choice time Mean ± SE (s)	Replicates	Choice time Mean ± SE (s)	Replicates		
Arbequina	Visual + olfactory	268 ± 30	25	255 ± 70	5	0,0327	0,8579^ns^
	Olfactory	336 ± 25	24	208 ± 64	6	4,7747	0,0374^**∗**^

Frantoio	Visual + olfactory	268 ± 28	23	218 ± 43	7	0,7723	0,3870^ns^
	Olfactory	158 ± 26	22	125 ± 48	8	0,4192	0,5227^ns^

Leccino	Visual + olfactory	346 ± 29	22	308 ± 45	8	0,4642	0,5013^ns^
	Olfactory	222 ± 35	23	166 ± 63	7	0,5883	0,4495^ns^

Within a row, the asterisk indicates a significant difference (*P* < 0.05).

ns: not significant.

SE: standard error.

**Table 2:** Number of antennal drumming series performed by *Psyttalia concolor* females during searching behavior on healthy and *Bactrocera oleae*-infested olives in two-choice bioassay in still air arena.

Cultivar	Treatment	Infested olives		Healthy olives		*F*	*P* value
		Drumming series Mean ± SE (N)	Replicates	Drumming series Mean ± SE (N)	Replicates		
Arbequina	Visual + olfactory	5,6 ± 1,1	25	1,0 ± 0,6	5	3,4609	0,0734^ns^
	Olfactory	7,0 ± 0,9	24	2,5 ± 1,3	6	5,3001	0,0209^*∗*^

Frantoio	Visual + olfactory	7,9 ± 1,3	23	1,6 ± 0,8	7	6,833	0,0142^*∗*^
	Olfactory	2,3 ± 0,3	22	1,5 ± 0,4	8	1,6977	0,2032^ns^

Leccino	Visual + olfactory	9,8 ± 1,6	22	1,4 ± 0,5	8	9,6881	0,0042^*∗*^
	Olfactory	2,1 ± 0,5	23	0,1 ± 0,1	7	5,531	0,0259^*∗*^

Within a row, the asterisk indicates a significant difference (*P* < 0.05).

ns: not significant.

SE: standard error.

**Table 3:** Principal component identified after Principal Component Analysis (PCA) of volatile emissions from three olive cultivars. Bolded components were analyzed using a General Linear Model to determine source of variation.

Principal component	Eigenvalue	Percentage	Cumulative percentage
**1**	**195.168**	**20.986**	**20.986**
**2**	**136.287**	**14.654**	**35.640**
**3**	**108.109**	**11.625**	**47.265**
**4**	**65.221**	**7.013**	**54.278**
**5**	**62.379**	**6.707**	**60.985**
**6**	**53.423**	**5.744**	**66.730**
7	50.994	5.483	72.213
8	43.666	4.695	76.908
9	39.679	4.267	81.175
10	35.437	3.810	84.985
11	33.083	3.557	88.542
12	25.223	2.712	91.255
13	21.784	2.342	93.597
14	20.431	2.197	95.794
15	17.363	1.867	97.661
16	11.942	1.284	98.945
17	0.9813	1.055	100.000

**Table 4:** Volatiles identified after discriminant analysis. Positive correlations with Canonical1 indicate volatiles representative of healthy fruits, while negative correlations compounds are expressive of infested olives.

Compound	Correlation with Canonical1
6-Methyl-3-methylene-5-hepten-2-one	0,15583672
Dihydromyrcenol	−0,316570777
Terpinolene	−0,446911371
Methyl carvacrol	−0,759893869
Linalool acetate	−0,445435614
2,6,11-Trimethyldodecane	0,421648972
Cyclosativene	−0,355430074
(*E,E*)-*α*-Farnesene	−0,535648427
Liguloxide	−0,117965501
1-Hexadecene	−0,21094794
*trans*-Methyl dihydrojasmonate	−0,279958519
