# Supplementary material for: VOCs-Mediated Location of Olive Fly Larvae by the Braconid Parasitoid Psyttalia concolor: A Multivariate Comparison among VOC Bouquets from Three Olive Cultivars
Source: Biomed Res Int. 2016 Feb 18;2016:7827615. doi: 10.1155/2016/7827615 (PMC4775789; doi:10.1155/2016/7827615)
Supplement: Supplementary file 1 — Tables S1, S2, and S3 present the volatiles emitted by healthy and olive fruit fly-infested fruits from cultivar Arbequina, Frantoio and Leccino, respectively. Table S4 showed the General Linear Model results. (A) Classes of volatiles emitted by healthy and infested olive fruits. (B) Effect test results for the two main variables (Cultivar and Infestation Status) and their interaction (Cultivar∗ Infestation Status).Table S5 provides the Eigenvectors of each volatile compounds emitted by olive fruits for six selected Principal Components. Table S6 highlighted the correlations between volatiles from olive fruits and factors identified after Multi-Factorial Analysis (MFA). Table S7 focuses on the effect tests of two way General Linear Models of Factors obtained after Multi-Factorial analysis (MFA) respect to the main variable (Cultivar and Infestation Status) and their interaction (Cultivar∗ Infestation Status). [file 7827615.f1.zip › Supplementary Table S5.doc]

**Supplementary Table S5. Eigenvectors of each volatile compounds emitted by olive fruits for 6 selected Principal Components.**

| **Compound** | **PC1** | **PC2** | **PC3** | **PC4** | **PC5** | **PC6** |
| --- | --- | --- | --- | --- | --- | --- |
| 6-methyl-3-methylene-5-hepten-2-one | -0.562110 | -0.278098 | 0.124406 | -0.335786 | 0.392320 | -0.177243 |
| octanal | -0.469379 | 0.327837 | -0.150943 | -0.531213 | 0.148616 | -0.293772 |
| isocineole | -0.556500 | 0.514247 | 0.066577 | 0.039811 | 0.176598 | 0.579812 |
| β-pinene | 0.236344 | -0.020166 | -0.664638 | -0.069570 | -0.501314 | 0.276281 |
| *o*-cymene | -0.493514 | 0.415020 | 0.000895 | -0.095994 | 0.303739 | 0.435258 |
| (*Z*)-3-hexenyl acetate | 0.814446 | 0.295600 | 0.073765 | 0.006193 | 0.329520 | -0.055684 |
| limonene | 0.020576 | 0.252056 | 0.004322 | -0.261287 | 0.109567 | 0.596131 |
| (*Z*)-β-ocimene | 0.652300 | 0.092937 | 0.459664 | -0.120210 | 0.250641 | -0.027506 |
| (*E*)-β-ocimene | 0.228136 | 0.463835 | 0.365574 | 0.089226 | 0.053237 | 0.149884 |
| γ-terpinene | -0.557706 | 0.518088 | 0.068347 | 0.048251 | 0.176893 | 0.570341 |
| dihydromyrcenol | -0.457986 | 0.489944 | -0.087205 | 0.182985 | 0.065002 | 0.543894 |
| terpinolene | -0.552762 | 0.517603 | 0.074521 | 0.061129 | 0.156448 | 0.580021 |
| *n-*undecane | 0.366223 | -0.037361 | 0.559135 | -0.098979 | -0.170781 | 0.071577 |
| nonanal | -0.553791 | 0.215021 | -0.278044 | -0.423183 | 0.213200 | -0.157015 |
| 2-methyl-6-methylene-1,7-octadien-3-one | -0.319358 | 0.069219 | 0.763925 | -0.019548 | 0.396736 | -0.075581 |
| neo-menthol | -0.834151 | -0.236682 | 0.001381 | 0.063616 | -0.007520 | -0.198531 |
| menthol | -0.534297 | 0.450759 | 0.004592 | 0.097400 | 0.302031 | 0.216025 |
| camphor | -0.011999 | -0.021553 | 0.639251 | -0.058118 | -0.565766 | 0.017308 |
| *n-*dodecane | 0.560077 | 0.185266 | 0.444137 | -0.464896 | -0.058028 | 0.214299 |
| decanal | -0.690142 | 0.011523 | -0.149888 | -0.504192 | -0.002656 | -0.191685 |
| dihydrocitronellol | 0.563645 | 0.110540 | 0.182231 | 0.173217 | -0.365762 | 0.355345 |
| methyl carvacrol | 0.193809 | 0.653268 | 0.444653 | 0.030045 | 0.073115 | 0.099728 |
| linalool acetate | 0.486285 | 0.143826 | 0.537298 | -0.205520 | -0.079439 | 0.046294 |
| (*E*)-2-decenal | -0.318317 | 0.258007 | 0.317397 | -0.144706 | -0.400200 | -0.290034 |
| 1-decanol | -0.349874 | 0.267425 | 0.332144 | 0.027633 | -0.122901 | 0.298337 |
| citronellyl formate | 0.180904 | -0.375816 | 0.060184 | -0.062420 | -0.159207 | 0.545271 |
| isobornyl acetate | -0.091431 | 0.695634 | -0.130913 | 0.092290 | -0.358639 | -0.042743 |
| 2-butyl-1-octanol | -0.044056 | 0.004122 | 0.567575 | -0.403871 | -0.314917 | 0.037829 |
| *cis*-2-tert-butylcyclohexyl acetate | -0.552214 | 0.197018 | 0.103392 | 0.078116 | -0.199569 | -0.336708 |
| *trans*-2-tert-butylcyclohexyl acetate | 0.513800 | 0.501273 | -0.169534 | -0.478485 | 0.137218 | -0.179672 |
| *n-*tridecane | -0.267874 | 0.154522 | 0.675297 | -0.291744 | 0.101492 | 0.053312 |
| undecanal | -0.353095 | 0.030423 | 0.451396 | 0.138735 | 0.429840 | -0.344090 |
| (*Z*)-2-tridecene | -0.420292 | 0.745045 | 0.247194 | -0.236652 | 0.021655 | -0.101734 |
| 2,6,11-trimethyldodecane | 0.248074 | -0.299937 | -0.318112 | -0.429913 | -0.004852 | 0.423887 |
| farnesane | -0.531679 | -0.205869 | 0.594807 | 0.001077 | 0.158143 | 0.229992 |
| 6-methyltridecane | 0.100798 | -0.710367 | 0.605235 | -0.168668 | -0.044460 | 0.104335 |
| heptylcyclohexane | -0.633681 | 0.262603 | 0.339921 | -0.060052 | -0.207039 | -0.039976 |
| α-terpynil acetate | -0.407454 | 0.400949 | -0.021189 | 0.180961 | 0.090239 | -0.135581 |
| cyclosativene | 0.454382 | -0.575049 | 0.612106 | 0.001737 | 0.189615 | 0.048260 |
| 3-hydroxy-2,4,4-trimethylpentyl 2-methylpropanoate | -0.585288 | 0.542516 | 0.074137 | 0.244163 | 0.157347 | 0.355986 |
| α-copaene | 0.616184 | -0.608661 | 0.286223 | 0.143149 | 0.178231 | 0.007433 |
| 1-undecanol | 0.083829 | 0.078814 | 0.646018 | -0.099931 | 0.269951 | -0.161307 |
| 7-epi-sesquithujene | 0.332914 | -0.047139 | 0.137246 | 0.589822 | 0.413284 | -0.094401 |
| 1-tetradecene | -0.483287 | 0.057125 | 0.520149 | -0.105992 | -0.102130 | -0.053338 |
| *n-*tetradecane | 0.465887 | 0.327089 | 0.530227 | -0.478233 | -0.165234 | 0.211261 |
| longifolene | -0.403830 | 0.378878 | -0.054508 | 0.136928 | 0.107386 | -0.194442 |
| α-cedrene | -0.425173 | -0.172545 | 0.287697 | 0.198528 | 0.354425 | 0.058664 |
| dodecanal | 0.299979 | 0.719958 | -0.121648 | 0.011227 | -0.324237 | -0.246157 |
| *cis*-α-bergamotene | 0.781517 | 0.295336 | 0.249662 | 0.020359 | 0.374068 | -0.011867 |
| β-caryophyllene | 0.644807 | -0.492084 | -0.040961 | -0.206674 | 0.095626 | 0.401550 |
| *trans*-α-bergamotene | 0.844347 | 0.402114 | 0.085403 | 0.063429 | 0.160679 | -0.051383 |
| *cis*-β-farnesene | 0.622005 | -0.049799 | 0.069089 | 0.352008 | -0.252824 | -0.351901 |
| α-humulene | 0.838082 | 0.220527 | -0.448591 | -0.029269 | -0.097519 | 0.154669 |
| geranylacetone | -0.234561 | 0.049205 | 0.539926 | -0.003248 | 0.576448 | -0.315372 |
| *trans*-β-farnesene | 0.848668 | 0.262466 | -0.156944 | -0.064223 | -0.015944 | 0.175479 |
| α-acoradiene | 0.220345 | 0.382420 | -0.110019 | 0.826022 | 0.066949 | 0.040667 |
| butylated hydroxyanisole | 0.269556 | -0.664161 | 0.587491 | -0.081941 | 0.079590 | 0.076230 |
| γ-muurolene | 0.451975 | 0.136561 | -0.375143 | -0.404522 | 0.242440 | 0.080262 |
| germacrene D | 0.642267 | 0.151573 | -0.366407 | 0.323991 | 0.310727 | -0.119197 |
| 1-dodecanol | -0.168241 | -0.299707 | 0.159309 | 0.398763 | -0.016923 | -0.160646 |
| β-selinene | -0.196873 | -0.204427 | 0.275853 | 0.356305 | -0.237968 | 0.255524 |
| valencene | 0.334908 | 0.470701 | 0.361315 | 0.245718 | -0.109516 | -0.178732 |
| viridiflorene | -0.208491 | -0.217431 | 0.329102 | -0.068476 | -0.203180 | 0.223966 |
| α-selinene | -0.357874 | 0.244891 | 0.250963 | 0.410703 | -0.319055 | -0.343854 |
| epi-cubebol | 0.082746 | -0.304703 | -0.244861 | -0.041668 | 0.165639 | -0.058957 |
| 6,9 dimethyltetradecane | 0.631167 | 0.180580 | 0.285455 | 0.083081 | -0.025211 | 0.008345 |
| *n-*pentadecane | 0.552169 | 0.094986 | 0.560980 | 0.043492 | 0.299972 | 0.273110 |
| (*E,E*)-α-farnesene | 0.186318 | 0.762315 | -0.029563 | 0.125788 | -0.149270 | 0.013537 |
| butylated hydroxytoluene | 0.250522 | 0.295224 | 0.483240 | 0.253861 | 0.187749 | -0.305953 |
| β-sesquiphellandrene | 0.404814 | 0.179754 | 0.271118 | 0.548120 | 0.086235 | -0.002429 |
| *cis-*dracunculifolol | -0.106120 | -0.699040 | 0.212477 | 0.203867 | 0.032324 | 0.039073 |
| lilal | -0.101030 | 0.614477 | -0.046904 | 0.008240 | -0.234948 | -0.154940 |
| liguloxide | 0.192743 | -0.454017 | 0.204721 | 0.459731 | 0.081524 | 0.083528 |
| 2,6,10-trimethyltetradecane | -0.195570 | 0.232591 | 0.657598 | 0.066145 | -0.390058 | -0.086943 |
| citronellyl butyrate | -0.402451 | 0.062910 | 0.460771 | 0.229529 | -0.546959 | -0.001192 |
| epi-longipinanolo | -0.094556 | -0.468832 | 0.425981 | 0.119438 | -0.321895 | 0.035159 |
| 5,8-diethyldodecane | -0.162651 | -0.178684 | 0.214976 | 0.454824 | -0.396594 | 0.137510 |
| 1-tridecanol | 0.491869 | 0.081054 | 0.138338 | 0.032797 | -0.255762 | -0.243370 |
| 1-hexadecene | 0.226020 | -0.217871 | -0.074371 | 0.358905 | 0.367753 | 0.466281 |
| *n-*hexadecane | 0.573694 | 0.358753 | 0.383404 | -0.293625 | -0.144570 | -0.297464 |
| tetradecanal | -0.543603 | 0.399449 | -0.105484 | -0.006587 | 0.258030 | 0.042360 |
| (*Z*)-7-hexadecene | 0.835185 | 0.275185 | -0.102207 | 0.095448 | 0.223734 | -0.040926 |
| epi-cedrol | 0.223982 | 0.723625 | 0.049002 | 0.133484 | -0.328490 | 0.074964 |
| epoxy-alloaromadendrene | -0.148363 | -0.771053 | 0.232308 | -0.171875 | 0.039237 | 0.088084 |
| 2,6,10-trimethylpentadecane | -0.189663 | 0.604275 | 0.323792 | -0.302377 | 0.017396 | -0.106626 |
| *trans-*methyl dihydrojasmonate | -0.162290 | 0.523719 | 0.131522 | 0.531402 | 0.121075 | 0.093655 |
| himachalol | -0.010164 | -0.102069 | 0.092989 | -0.451105 | 0.282746 | -0.169808 |
| α-bisabolol | 0.355548 | 0.378120 | 0.046923 | -0.038698 | -0.162794 | 0.242619 |
| 1-tetradecanol | 0.280916 | -0.004307 | 0.029161 | -0.147301 | -0.718375 | 0.171752 |
| 1-heptadecene | 0.248711 | 0.364054 | -0.370214 | 0.089536 | 0.315599 | -0.299355 |
| 3,5-di-tert-butylpyrocatechol | 0.796283 | 0.317959 | 0.313056 | -0.044894 | 0.329674 | 0.010286 |
| *n-*heptadecane | 0.580512 | 0.510649 | 0.158317 | -0.438898 | 0.041123 | -0.020259 |
| pentadecanal | -0.699717 | -0.014149 | 0.190575 | 0.038666 | 0.244335 | 0.098024 |
